# Electron Channeling Contrast Imaging of Ferroelastic Domains

**DOI:** 10.1002/adma.202515762

**Published:** 2026-01-21

**Authors:** Wei Peng, Aditya Singh, M. Haroon Qaiser, Philipp Fahler‐Muenzer, Freya Watson, Liang Si, Yining Xie, Katarzyna E. Sopińska, Daniel A. Chaney, Ana M. Sanchez, T. Ben Britton, Richard Beanland, Marios Hadjimichael, Marin Alexe

**Affiliations:** ^1^ Department of Physics University of Warwick Coventry UK; ^2^ College of Materials Science and Engineering Hunan University Changsha China; ^3^ Department of Materials Engineering University of British Columbia Vancouver Canada; ^4^ School of Physics Northwest University Xi'an China; ^5^ ESRF–The European Synchrotron Grenoble France

**Keywords:** domain imaging, electron microscopy, ferroelasticity, ferroelectricity

## Abstract

Ferroelastic phase transitions lead to the formation of ferroelastic twins, resulting in spatial inhomogeneities in the crystal structures and functional properties of ferroelastic materials. Despite the importance of such nanoscale twins in oxide heterostructures, their direct, non‐invasive observation has relied on cumbersome and low‐throughput techniques such as transmission electron microscopy and synchrotron X‐ray diffraction, or been restricted to materials with coexistence of ferroelectric and ferroelastic states, by detecting the coupled ferroelectric order. In this study, the application of electron channeling contrast imaging (ECCI) in a scanning electron microscope for imaging ferroelastic domains in various oxide heterostructures is demonstrated. These include systems where ferroelasticity is coupled with critical functional properties such as ferroelectricity, magnetism and charge transport. The versatility of this imaging method is highlighted across different heterostructure geometries, from bare thin films to multilayers, achieving an impressive resolution of 6 nm. ECCI presents a powerful approach for exploring the rich spectrum of ferroelasticity‐coupled phenomena in oxide heterostructures.

## Graphical abstract

A simple scanning electron microscopy technique, electron channeling contrast imaging, unveils nanoscale ferroelastic domains and their coupling to functional properties in oxide heterostructures. The work demonstrates high‐resolution mapping of both surface and buried domains, broadening the experimental toolbox for ferroic and quantum oxide systems.

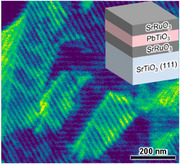

## 1 Introduction

Ferroelasticity, the largest class of ferroic properties, is characterized by elastic hysteresis arising from the switching between multiple crystallographic orientations (twins) under applied mechanical stress [1, 2]. Unlike ferroelectricity and ferromagnetism, ferroelasticity does not depend on specific electronic orbital occupations and symmetry breaking. As a result, it is widely compatible with other key material properties, such as polarity, magnetism and charge transport [3, 4, 5, 6, 7, 8]. In transition metal oxides, where structural and electronic degrees of freedom are strongly intertwined, ferroelasticity can significantly modulate electronic behavior. The formation of ferroelastic domains introduces structural heterogeneity and inhomogeneous strain, alters material anisotropy [5, 9, 10, 11, 12], and gives rise to 2D topological defects, namely domain walls, which have been associated with emergent functionalities [3, 6, 13, 14, 15, 16].

In oxide heterostructures—key platforms for studying such phenomena—the characteristic size of ferroelastic domains can be reduced to the nanometer scale following a thickness scaling law [17, 18], dramatically increasing domain density and amplifying their influence. Accordingly, spatial imaging of nanoscale ferroelastic domains is essential for investigating structural heterogeneity and the correlation with electronic, magnetic, or transport phenomena.

Transmission electron microscopy (TEM) and scanning transmission electron microscopy (STEM) provide direct visualization of strain and crystalline structure at atomic resolution, making them effective probes of ferroelastic order [6, 19]. However, these methods are inherently local, typically offering a field of view (FOV) limited to a few hundred nanometers, which hampers the study of large‐area domain distributions or mesoscale structural variations in complex oxides. Additionally, the invasive and time‐consuming sample preparation required for TEM/STEM limits their flexibility and throughput. X‐ray diffraction (XRD), particularly when implemented with high‐flux, nano‐focused and/or coherent beams, can also resolve ferroelastic domain structures [20, 21, 22]. Yet such measurements necessitate synchrotron radiation sources, constraining their accessibility and adaptability. Alternatively, in ferroelectric systems, ferroelastic domains can sometimes be inferred indirectly through techniques sensitive to coupled orders, such as piezoelectric force microscopy (PFM) [23] or second harmonic generation (SHG) [24]. Nonetheless, these methods are limited to materials exhibiting inversion symmetry breaking such as ferroelectrics. There remains a critical need for a high‐throughput, non‐invasive imaging method broadly applicable across a range of ferroelastic materials—whether metallic or insulating, polar or non‐polar—to accelerate the understanding and engineering of ferroelasticity‐related functionalities. This method must resolve structural domains with orientation differences as small as 0.1°, while offering structural sensitivity down to a few nanometers in both the in‐plane and out‐of‐plane directions of the film.

Electron channeling contrast imaging (ECCI) in a scanning electron microscope (SEM) offers such a pathway [25]. The technique is based on the collection of backscattered electrons (BSEs) which can vary according to the crystal structure and structural orientation. Over the past few decades, ECCI has proved effective in visualizing crystalline grains, twins, dislocations, and grain boundaries [26, 27, 28, 29]. However, its potential for imaging ferroelastic domains in epitaxial oxide heterostructures, which have relatively small sample volumes for electron scattering, has been largely unexplored [30]. In this work, we demonstrate the efficacy of ECCI to resolve ferroelastic domain structures across a range of oxide thin films, including ferroelectrics and ferromagnetic metals, with high sensitivity and lateral resolution down to 6 nm. Compared to the aforementioned techniques, ECCI offers a good combination of rapid acquisition, non‐invasiveness, and minimal sample preparation. Once key parameters, such as sample tilting, rotation, beam current, and voltage, are optimized, high‐quality domain images can be acquired within minutes with a typical FOV of ∼100 µm. These attributes position ECCI as a powerful and versatile tool for investigating ferroelastic order and associated emergent phenomena that may have previously remained hidden.

## 2 Observation of Ferroelastic Domains by ECCI

**FIGURE 1:**
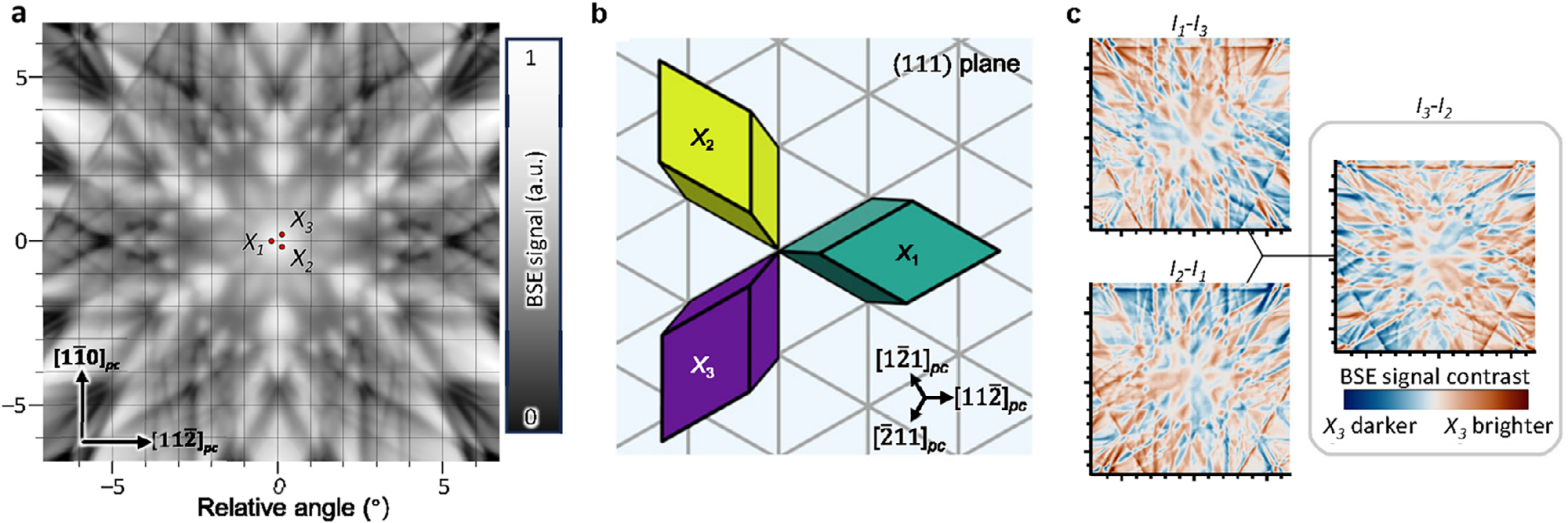
Mechanism of electron channeling contrast in (111)*_pc_* SrRuO_3_. (a) Simulated electron channeling pattern (ECP) from a (111)*_pc_* SrRuO_3_ single crystal in the absence of ferroelastic domains with a 10  keV primary electron beam. The color scale shows the simulated backscattered electron (BSE) signal, normalized to its maximum value. a.u.: arbitrary units. Brighter regions correspond to specimen orientations that generate more BSEs. The horizontal and vertical scales represent the angle of the incident electron beam relative to the specimen along the ${\left[11\bar{2}\right]}_{pc}$ and ${\left[1\bar{1}0\right]}_{pc}$ directions respectively, with the overlaid mesh indicating a specimen tilt of 1°.(b) Schematic geometry of the three ferroelastic variants of SrRuO_3_ (*X_1_*, *X_2_*, *X_3_*) with respect to the (111) plane of SrTiO_3_ in an epitaxial heterostructure. The [111]*_pc_* directions of these ferroelastic variants tilt by approximately 0.1° away from the [111] direction of SrTiO_3_ along the ${\left\langle 11\bar{2}\right\rangle }_{pc}$ directions. The three dots in (a) mark the position of the actual center of the ECP for each domain variant, as denominated. As demonstrated in the supplementary interactive simulation [32], the three points move together with the specimen tilting. (c) Maps of the contrast between pairs of domains over the same range of specimen tilts as in (a). The maps are calculated by shifting the bulk SrRuO_3_ ECP by 0.1° in the ${\left\langle 11\bar{2}\right\rangle }_{pc}$ directions corresponding to the marked (*X*_1_, *X*_2_, *X*_3_) points in (a). The BSE signal corresponding to each domain is denoted as *I*_1_, *I*_2_, and *I*_3_, respectively, and the difference maps shown here are calculated as *I*_1_ − *I*_3_, *I*_2_ − *I*_1_, and *I*_3_ − *I*_2_. For instance, regions of red color in the plot of *I*_3_ − *I*_2_ (far right) show that, at those tilt angles, domain *X_3_* is brighter than *X_2_*.

In SEM, the primary electron beam penetrates the sample and is scattered, primarily by inelastic events. However, a fraction of these electrons retains energies nearly identical to the primary beam and is coherently scattered by the atomic lattice, propagating within the crystal as Bloch waves. The paths and interference of these Bloch waves are extremely sensitive to the crystal's orientation relative to the incident beam, so even minuscule angular deviations dramatically alter their propagation and subsequent escape as BSEs^31^. As an example, Figure 1a shows a simulated [111]*_pc_* electron channeling pattern (ECP) for SrRuO_3_, using a 10 keV primary electron beam (for simplicity, we use pseudocubic indexing, indicated by the subscript *pc*). SrRuO_3_ is a perovskite oxide with typical ferroelastic domains that originate from its small orthorhombic structural distortion [11]. The horizontal and vertical axes in Figure 1a indicate the angle between the electron beam and the specimen along the ${\left[11\bar{2}\right]}_{pc}$ and ${\left[1\bar{1}0\right]}_{pc}$ directions respectively, with an intensity corresponding to the backscattered electron signal collected at that relative angle. In practice, the direction of the incident beam is fixed, and the specimen is tilted (Figure S1); an overlaid grid at intervals of 1° indicates specimen tilt angle. Thus, bright regions correspond to specimen orientations that enhance backscattering, whereas dark regions mark angles where the wave propagates more deeply, producing fewer BSEs. The fraction of BSEs carrying this crystallographic contrast is relatively small, and its effect is most easily detected in the absence of topographical and compositional variations.

By growing a SrRuO_3_ film coherently on (111) SrTiO_3_, SrRuO_3_ presents three ferroelastic variants (*X*_1_, *X*_2_, *X*_3_, Figure 1b), related by the substrate's threefold symmetry [11]. The thin film divides into domains, each with a [111]*_pc_* direction tilted by approximately 0.1° from the surface normal, and each variant therefore presents a slightly different orientation to the incident electron beam. The BSE yield for each domain can be determined from the ECP, where the backscattered intensity for each of the three orientations is given by points at the vertices of an equilateral triangle (Figure 1a). The relative positions of these points are fixed by the different orientations of the three variants and if the sample is tilted, the full ECP pattern is shifted. Therefore, the three points corresponding to domains *X*_1_, *X*_2_, and *X*_3_ move across the pattern without changing their relative geometry.

Contrast between two domains will be found when one point corresponds to an incident beam direction with a backscattered yield that is different to the second. To estimate this relative contrast, we consider the three cases in which the ECP corresponds to the *X*_1_, *X*_2_, and *X*_3_ domains which generate the respective BSE signals *I*_1_, *I*_2_, and *I*_3_. The relative contrast between each pair of variants, calculated as *I*_1_ − *I*_3_, *I*_1_ − *I*_2_, and *I*_2_ − *I*_3_ is mapped in Figure 1c. To obtain good contrast between all three domains, the points on the ECP must all have different intensities. The magnitude of this three‐domain contrast (|*I*_1_ − *I*_3_| + |*I*_2_ − *I*_1_| + |*I*_3_ − *I*_2_|) is presented in Figure S1 and can be investigated using the supplementary interactive simulation [32]. Although some contrast is detectable at almost any angle, it strengthens at sample tilts above 4°. Pinpointing sample orientations that give optimal contrast requires a precision in specimen tilt of 0.1° or less (as might be expected when differences in structural orientation of 0.1° are being examined). The consequence of this high sensitivity to specimen orientation is that a practical approach is empirical: orientations of maximal channeling are located using ECPs or low‐magnification backscattered electron images, then specimen tilt/rotation is fine‐tuned in ∼0.1° increments to maximize contrast between domains.

Monte Carlo simulations (CASINO; see Figure S1) confirm that, at 10 keV, most BSE interactions occur within the top 50 nm of the SrRuO_3_ layer [33]. In reality, the interaction volume associated with channeling interaction contrast is likely even smaller than the continuous‐slowing‐down approximation predicts [34], so ECCI may be viable on thinner films. Practically, optimal contrast demands a highly coherent, low‐convergence primary beam (<0.1°) and sufficient beam current, balanced against the need for a wide‐angle BSE detector to boost collection efficiency. These factors often conflict—narrower convergence improves channeling sensitivity but reduces current—so each microscope–sample combination requires a tailored compromise.

### 2.1 Ferroelastic Domains in a Magnetic Metal

**FIGURE 2:**
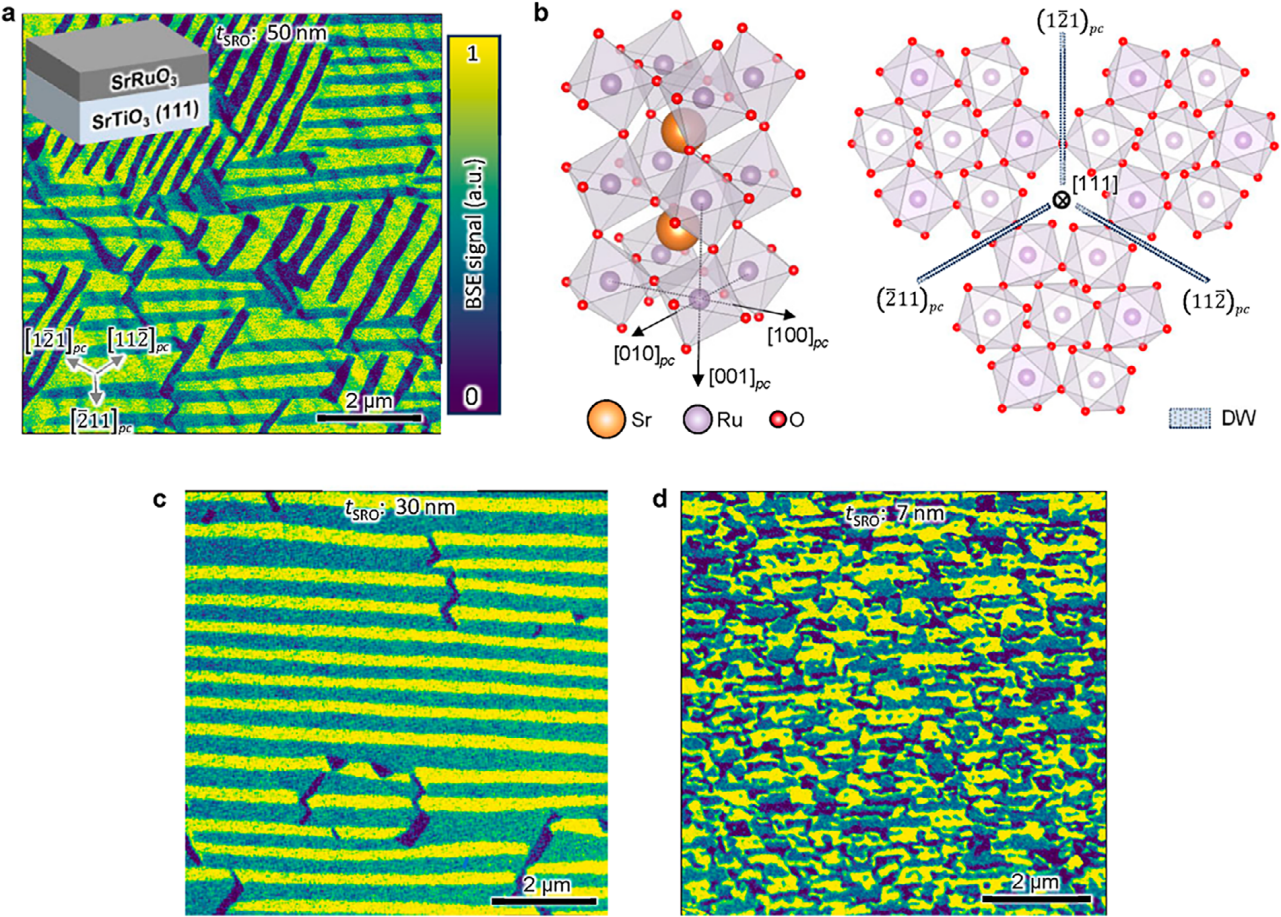
Imaging ferroelastic domains in (111)*_pc_* SrRuO_3_. (a) ECCI micrograph of a 50 nm‐thick SrRuO_3_ film, revealing three ferroelastic variants. The image was post‐processed in false colors, with color scale indicating the BSE signal normalized to its maximum value (the same color scale is used throughout the text). Inset shows the schematic heterostructure. (b) Crystal structure of SrRuO_3_ at room temperature and schematic of the domain wall orientations in the projected (111) crystalline plane (Sr atoms are omitted for better visualization of the oxygen octahedra). The different ferroelastic domains show clearly different propagation patterns of oxygen octahedral tilting. (c,d) ECCI micrographs of 30 nm‐ and 7 nm‐thick SrRuO_3_ films, respectively, showing different domain morphologies. The crystalline orientations of these micrographs are the same as that in (a).

To test the applicability of ECCI for domain mapping, we begin with a coherently strained, 50 nm‐thick SrRuO_3_ (111)*_pc_* film (Figures S2 and S3). The ECCI micrograph in Figure 2a (shown in false colors, the raw image was presented in Figure S4) exhibits three distinct contrasts with nearly equal populations, consistent with the predicted number of ferroelastic domain variants. These domains form stripe‐like microtextures, with a width of approximately 260 nm and domain walls aligning along the {$11\bar{2}$}*_pc_* plane. Figure 2b illustrates the orthorhombic structure of SrRuO_3_ and its variation between domains, which is mainly manifested in the change of oxygen octahedral tilting. This is supported by our density functional theory (DFT) calculations (see Methods and Figure S5).

As the film thickness decreases to 30 nm, the ferroelastic domains in SrRuO_3_ transform into a stripe pattern predominantly oriented in a single direction (Figure 2c). The domain width is approximately 210 nm. A comparison of the surface topography (Figure S2) with the ECCI micrograph suggests the correspondence between the domain orientation and topographic step terraces imprinted from substrate's miscut. Further reducing the SrRuO_3_ thickness to 7 nm results in scattered ferroelastic domains with the domain width decreasing to approximately 100 nm (Figure 2d). The evolution of domain morphology signifies a type of thickness scaling. With the film thickness decreasing, the surface/interface boundary conditions are expected to dominate the thermodynamic and structural stability, often outweighing bulk elastic or electrostatic energies. Therefore, the unit‐cell step terraces and islands in thinner films (<30 nm) originated from the miscut substrate and film growth present an important type of boundary condition to modulate the nucleation and growth of ferroelastic domains. In addition, it is expected that local variation of the step terrace, such as bonding environment and step bunching, may disrupt the stripe textures, e.g., giving rise to dark minority domains in the gap between the other two dominant stripe domains in Figure 2c. More detailed investigations combining theoretical and experimental approaches are required to understand the dependence of domain morphology on a variety of factors, including film growth and boundary conditions.

SrRuO_3_ turns ferromagnetic below a Curie temperature of approximately 160 K and we have previously shown that the magnetic anisotropy is coupled to the ferroelastic ordering due to a significant spin‐orbit coupling [11, 35]. In Figure S6, we show that each ferroelastic species responds differently to a scanning magnetic field, resulting in complex anomalous Hall conduction with features resembling those of topological Hall effects. Direct observation of these domains will facilitate our understanding of magnetoelastic coupling and magnetotransport in magnetic oxides. Importantly, the thickness‐dependent domain evolution should be taken into consideration while evaluating the thickness‐dependent magnetic properties, including structurally pinned magnetic domains, magnetotransport and spintronic functionalities, and domain wall‐coupled structural/electronic reconstruction, which may have been overlooked before [9, 11, 36, 37, 38].

### 2.2 Ferroelastic Domains in Ferroelectric Films

**FIGURE 3:**
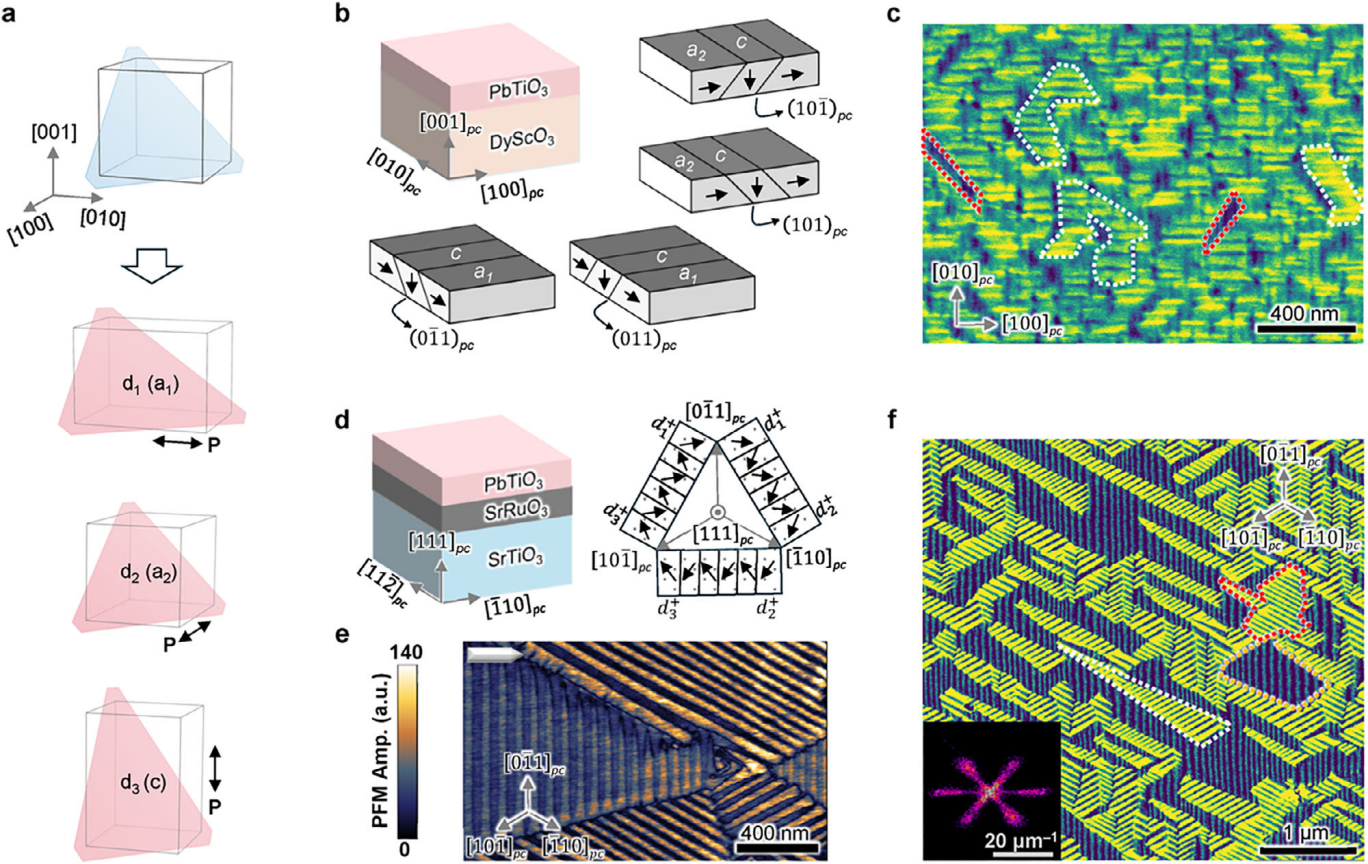
Imaging ferroelastic domains in bare ferroelectric PbTiO_3_ films. (a) Ferroelastic/ferroelectric phase transition of PbTiO_3_ from a cubic to a tetragonal structure upon cooling, which exhibits three orientation states, referred to as *d*_1_/*d*_2_/*d*_3_ in the (111)*_pc_*‐oriented heterostructure or *a*_1_/*a*_2_/*c* in the (001)*_pc_* heterostructure, with the polarization (*P*) coupled to the tetragonal long axis. The blue‐ and pink‐shaded planes indicate the (111)*_pc_* plane. (b) In a heterostructure of PbTiO_3_/DyScO_3_ (110) with a sufficient thickness of PbTiO_3_, the ferroelastic domains organize into *a*_1_/*c* and *a*_2_/*c* superdomains, with the domain walls in the $\left(011\right)/\left(0\bar{1}1\right)$ and $\left(101\right)/\left(10\bar{1}\right)$ planes, respectively. (c) ECCI micrograph of the heterostructure as in b), with a PbTiO_3_ thickness of approximately 58 nm. An *a*_1_/*c* superdomain and an *a*_2_/*c* superdomain are marked by white and red dashed lines, respectively. (d) In a heterostructure of PbTiO_3_/SrRuO_3_/SrTiO_3_ (111), the three variants of PbTiO_3_ with upward polarization, namely $d_{1}^{+}$, $d_{2}^{+}$, $d_{3}^{+}$, are predicted to form three superdomain species, comprising two variants arranged in a head‐to‐tail pattern and stacked along the ${\left\langle 11\bar{2}\right\rangle }_{pc}$ direction. (e) In‐plane PFM images of a 30 nm‐thick PbTiO_3_ (111)*_pc_* film, showing three ferroelectric/ferroelastic superdomain species with a periodicity of 60–70 nm. (f) ECCI micrograph of the PbTiO_3_/SrRuO_3_/SrTiO_3_ (111) heterostructure. The inset shows the corresponding FFT, from which a periodicity of approximately 65 nm can be extracted. White, orange and red dashed lines mark the *d*_1_/*d*_2_, *d*_2_/*d*_3_ and *d*_1_/*d*_3_ superdomains, respectively.

Ferroelectric oxides are another important class of materials, in which ferroelasticity plays an important role. We use PbTiO_3_, a prototypical ferroelectric oxide adopting a tetragonal structure at room temperature [39], to further showcase the applicability of ECCI. As shown in Figure 3a, the tetragonal unit cell has three possible orientations with spontaneous strain, corresponding to the ferroelastic variants of PbTiO_3_. We first choose a simple heterostructure of PbTiO_3_ grown on an orthorhombic DyScO_3_ (110) substrate (Figure 3b), the domain structures of which have been intensively studied by PFM, TEM and XRD [19, 40]. Along the (001)*_pc_* orientation—corresponding to (110) of the orthorhombic substrate—the long axis of tetragonal PbTiO_3_ is either in plane (referred to as *a*_1_ and *a*_2_ variants) or out of plane (referred to as *c* variant). It has been well established that, to minimize the elastic energy in sufficiently thick PbTiO_3_ films (e.g., 58 nm in the current study), the ferroelastic variants of PbTiO_3_ will self‐organize into superdomain bands, namely *a*_1_/*c* or *a*_2_/*c* twins with the domain walls in the {101}_*pc*_ planes (Figure 3b) [19, 20]. This is corroborated by the PFM images in Figure S7.

The secondary electron image acquired by SEM, a commonly used imaging mode that is sensitive to surface topography, failed to resolve any domain features (Figure S8). By contrast, the ECCI micrograph of this heterostructure in Figure 3c reveals approximately 50 nm wide stripe microtextures perpendicular to the [100]*_pc_* or [010]*_pc_* directions, in resemblance to the ferroelectric domain structures. However, due to a tilted geometry of the domain walls, the domains overlap while viewed along the out‐of‐plane direction (Figure 3b), resulting in blurred contrast between different domains in the ECCI micrograph. Additionally, depending on the orientation of the {101}_*pc*_ domain walls, the *c* and *a*_1_/*a*_2_ domains tilt in four different directions, potentially resulting in contrast variation among different *c* domain species, but it is challenging to resolve in the image [41]. These results suggest the effectiveness of ECCI for directly observing ferroelastic domains in oxide thin films. Importantly, it is robust against variation in sample surface conditions and features rapid acquisition times for an overall picture of the domains and structural heterogeneity. However, it does not determine the polarization directions (e.g., pointing up or down for the *c* domains).

Another thin film system of ferroelectrics that has recently attracted great interest is the (111)*_pc_*‐oriented tetragonal ferroelectrics for their complex domain structures along with peculiar response to electrical and mechanical excitations [42, 43, 44, 45]. In such a system, the three structural variants are rotation‐symmetric around the out‐of‐plane axis and energetically degenerate (thereby referred to as *d*_1_, *d*_2_, *d*_3_). This produces a complex free energy landscape that significantly modifies the behavior of the material, leading to intriguing functional properties. Motivated by this, we prepared a PbTiO_3_/SrRuO_3_/SrTiO_3_ (111) heterostructure (Figure 3d). The dissimilar top and bottom interfaces of PbTiO_3_ in such a heterostructure create asymmetric electrostatic boundary conditions and are expected to induce a built‐in electric field to imprint PbTiO_3_. Indeed, out‐of‐plane PFM confirms an upward self‐polarization state in the pristine film (Figure S9), i.e., *d*_1_^+^, *d*_2_^+^, and *d*_3_^+^.

The three polarization variants of PbTiO_3_ (111)*_pc_* are predicted to form three superdomain bands, each comprising two variants arranged in a head‐to‐tail pattern and stacked along the ${\left\langle 11\bar{2}\right\rangle }_{pc}$ direction, separated by the {110} planes (Figure 3d, ref [30, 46]). Figure 3e presents an in‐plane PFM image of the heterostructure with a PbTiO_3_ thickness of approximately 30 nm. The micrograph reveals a domain microtexture that is consistent with theoretical predictions, with a well‐defined domain periodicity of 60–70 nm within each superdomain. The ECCI micrographs in Figure 3f and Figure S10 corroborate this finding, showing an average domain periodicity of 65 nm, determined via fast Fourier transform (FFT). Furthermore, ECCI reveals similar domain microtextures with reduced domain sizes in thinner PbTiO_3_ (111)*_pc_* films, which is consistent with the PFM images (Figure S11). This result again verifies the effectiveness of ECCI for characterizing epitaxial ferroelectric thin films.

### 2.3 Buried Ferroelastic Domains in Ferroelectric Capacitors

**FIGURE 4:**
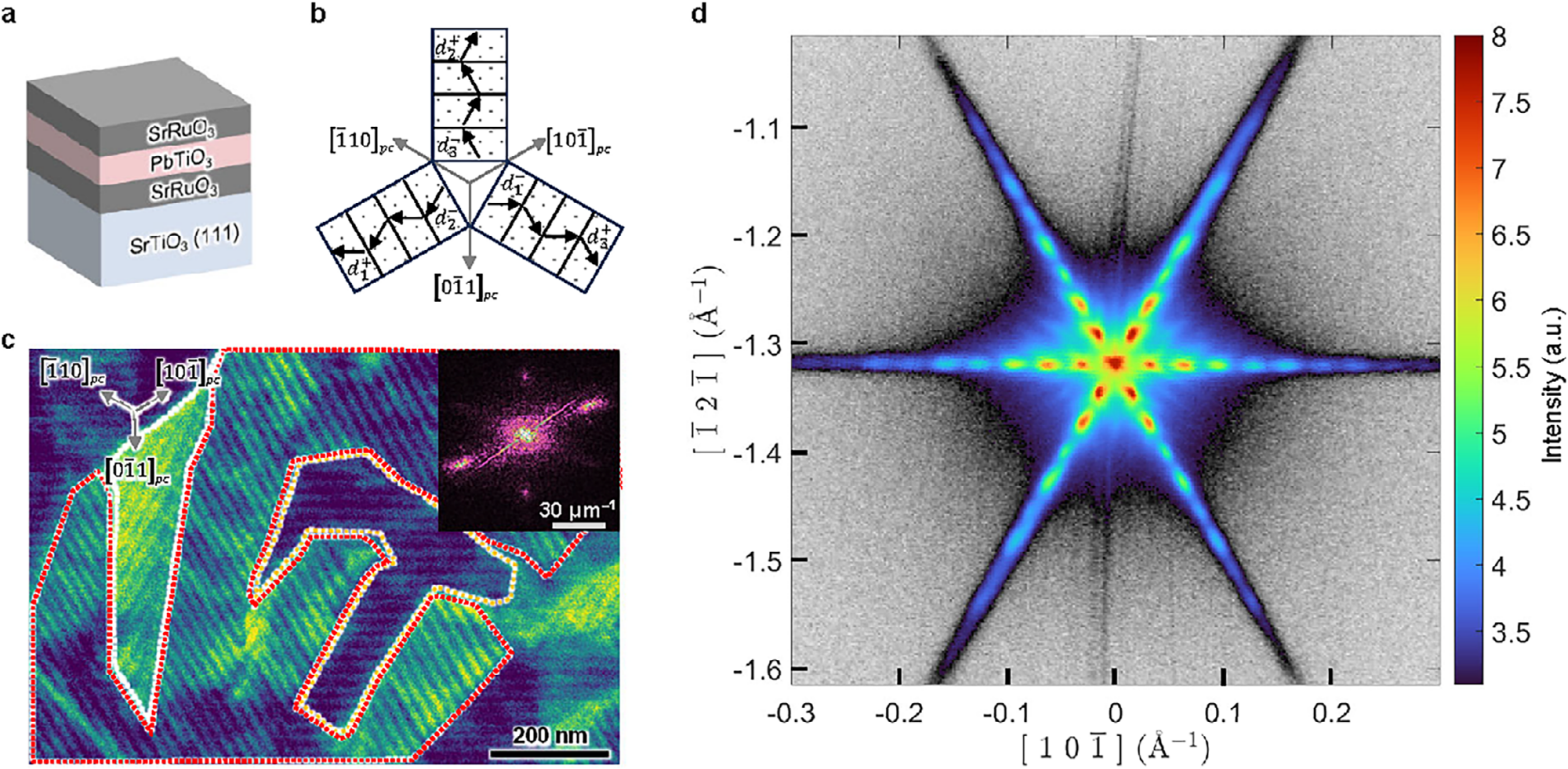
Imaging buried ferroelastic domains in ferroelectric PbTiO_3_ capacitors. (a) Symmetric capacitor of SrRuO_3_/PbTiO_3_/SrRuO_3_/SrTiO_3_ (111), which is expected to exhibit mixed up and down polarization variants. (b) Predicted ferroelectric domain structures for a (111)*_pc_*‐oriented tetragonal ferroelectric with mixed up/down polarization variants. Note that only *d*_1_^+^/*d*_2_^−^, *d*_2_^+^/*d*_3_^−^ and *d*_3_^+^/*d*_1_^−^ are shown here. The other three superdomain species, *d*_1_^−^/*d*_2_^+^, *d*_2_^−^/*d*_3_^+^, *d*_3_^−^/*d*_1_^+^, are symmetrically equivalent and thus omitted for simplicity. (c) ECCI micrograph of the heterostructure. The inset shows the corresponding FFT, from which a well‐defined domain periodicity of approximately 22 nm is deduced. White, orange and red dashed lines mark the *d*_1_/*d*_3_, *d*_2_/*d*_3_ and *d*_1_/*d*_2_ superdomains, respectively. (d) Section of a reconstructed reciprocal space plane showing the (101)*_pc_* peak of PbTiO_3_ with three‐fold‐rotation symmetric satellite peaks, which is consistent with the superdomain microtextures. The plane is perpendicular to [111]*_pc_* and centered on the (2/3 2/3 2/3)*_pc_* position. The reconstructed plane is shown using a hybrid color scale to clearly visualize additional scattered intensity in close proximity to the Bragg peak. High intensities are mapped to the logarithmic colorbar shown and all values below the minimum are expressed in linear gray scale.

A more practically relevant heterostructure for ferroelectrics is capacitors, which are fundamental components in various applications, including ferroelectric tunnel junctions [47] and energy storage devices [48]. Detailed knowledge of their domain structures is critical for unlocking their technological potential. Using SrRuO_3_/PbTiO_3_/SrRuO_3_/SrTiO_3_ (111) as an example (Figure 4a; Figures S12 and S13), we demonstrate that ECCI can probe the ferroelastic domains of the underlying PbTiO_3_ layer, even with a 3 nm‐thick SrRuO_3_ capping layer.

In such a symmetric capacitor, PbTiO_3_ experiences negligible built‐in electrostatic fields but a sizeable depolarization field due to imperfect screening of the SrRuO_3_ electrode, resulting in coexistence of up‐ and down‐polarization variants (*d*_1_^+^, *d*_2_^+^, *d*_3_^+^, *d*_1_^−^, *d*_2_^−^, and *d*_3_^−^, ref [49, 50, 51].). These variants are predicted to organize into superdomain bands to minimize the electrostatic and elastic energies, each comprising paired up‐ and down‐polarization variants (e.g., *d*_1_^+^ and *d*_2_^−^) arranged in a head‐to‐tail configuration along the ${\left\langle \bar{1}10\right\rangle }_{pc}$ directions, separated by $\left\{11\bar{2}\right\}$ planes (Figure 4b, ref [46]).

To investigate the domain structures, we first performed ECCI. The ECCI micrograph shows stripe‐like domain microtextures with a periodicity of approximately 22 nm (corresponding to a domain width of 11 nm), organized into superdomains along the ${\left\langle \bar{1}10\right\rangle }_{pc}$ directions (Figure 4c; Figure S12), which is consistent with the theoretical prediction. Additionally, large regions of contrast variation superimposed on the stripe subdomains within the same superdomain likely originate from the SrRuO_3_ layers. Based on simulations of backscattered electrons from such a heterostructure, PbTiO_3_ contributes the dominant signal owing to the large atomic mass of Pb atoms, in addition to minority signal from SrRuO_3_ (Figure S14). More impressively, we managed to resolve 6 nm‐wide stripe domains in an even thinner PbTiO_3_ film (∼9 nm) within the same capacitor structure, demonstrating the remarkable spatial resolution of ECCI (Figure S15).

PFM measurements on SrRuO_3_/PbTiO_3_/SrRuO_3_/SrTiO_3_ (111) are challenging due to the need for patterning the conductive capping layer with small lateral dimensions to minimize current leakage and enhance piezoresponse (see Methods). Furthermore, the small domain size (∼10 nm) approaches the resolution limit of PFM, rendering it ineffective for this system (Figure S16). However, the periodic domain microtextures are ideally suited to reciprocal space probes and we therefore employed grazing incidence synchrotron‐based XRD to investigate the domain structure as an alternative validation of the ECCI findings. Figure 4d shows a reconstructed 2D section of reciprocal space focused on the (101)*_pc_* peak of PbTiO_3_ (see Methods). Due to the periodic nature of the polarization and structural distortions, diffraction satellites arise near the Bragg peak. The three sets of satellite peaks, present along the ${\left\langle \bar{1}10\right\rangle }_{pc}$ directions, are consistent with the superdomain configurations in Figure 4b. Accordingly, a domain periodicity of approximately 20 nm was deduced, aligning closely with real‐space observation by ECCI.

The correspondence between ECCI and XRD results underscores the robustness of ECCI in resolving ferroelastic domain structures, even in complex heterostructures. However, due to the relative insensitivity of ECCI to up/down polarizations, we are unable to distinguish different polarization configurations, e.g., either *d*_1_^+^/*d*_2_^−^ or *d*_1_^−^/*d*_2_^+^. Besides, although our results are consistent with the predicted domain structures, we don't rule out possible existence of other configurations, such as *d*_1_^+^/*d*_1_^−^, which can also minimize the electrostatic energy. Future in‐depth studies combining other characterization techniques are required to uncover the detailed ferroelectric/ferroelastic domain structures.

The thickness limit of a capping electrode below which buried ferroelastic domains can be resolved is another issue for future investigations. The backscattered electron yield is determined by a few intrinsic material parameters, including atomic number, mass density and crystalline structure. For a multilayer heterostructure, the volume ratio between each layer determines the proportion of signal contributed by a targeted layer in the image and thus its resolvability. Besides, beam energy is a critical extrinsic parameter to determine the interaction volume. Therefore, these factors should be taken into careful consideration for the exploitation of ECCI. In light of this, we found that, for a SrRuO_3_/SrTiO_3_/PbTiO_3_/SrTiO_3_/DyScO_3_ heterostructure with 17 nm‐thick capping SrRuO_3_ and 2 nm‐thick buffer SrTiO_3_, the ferroelastic domains of PbTiO_3_ can be still resolved.

## 3 Conclusions

The present results showcase the versatility and sensitivity of ECCI for observing ferroelastic domains in a variety of thin film systems. In particular for the study of ferroelectric materials, an important playground of ferroelasticity, it would be significantly complementary to PFM, which is sometimes hindered by a reliance on electric boundary conditions of materials or quality of ancillary devices such as PFM tips. In addition, advancements in modern in‐situ SEM enable the application of external stimuli–such as temperature, stress, and electric fields–allowing real‐time observation of dynamic processes associated with ferroelastic switching. Moreover, it is worth noting that this technique is essentially a probe of the crystalline orientation states. Therefore, ECCI is expected to be beneficial for the study of materials systems where the crystalline orientation plays an influential role, e.g., 2D materials and Moiré superlattices [52].

## 4 Methods

### 4.1 Sample Growth

SrRuO_3_/SrTiO_3_, PbTiO_3_/SrRuO_3_/SrTiO_3_, and SrRuO_3_/PbTiO_3_/SrRuO_3_/SrTiO_3_ heterostructures were fabricated in a pulsed‐laser deposition system equipped with a KrF excimer laser (248 nm). A high‐pressure reflection high‐energy electron diffraction system was used to monitor the growth. The growth was performed under a constant oxygen pressure of 100 mTorr. Bottom SrRuO_3_ was grown with a laser fluence of 2.0 J cm^−2^ while PbTiO_3_ and top SrRuO_3_ were grown with a laser fluence of 1.2 J cm^−2^. Before deposition, SrTiO_3_ (111) substrates were etched with a buffered hydrofluoric acid solution and then annealed in air at 1000°C for 3 h to produce an atomically flat surface with a unit‐cell step terrace structure. For PbTiO_3_/DyScO_3_ heterostructures, PbTiO_3_ was grown under a constant oxygen pressure of 75 mTorr, with a laser fluence of 1.6 J cm^−2^. Before deposition, DyScO_3_ (110) substrates were annealed in oxygen at 1150°C for 4 h to produce an atomically flat surface with a unit‐cell step terrace structure.

### 4.2 ECP Simulation

Dynamical simulation of SrRuO_3_ Kikuchi pattern was generated from pattern simulation software ESPRIT DynamicS (Bruker Nano Analytics, Connecticut, USA). The electron beam voltage was set to match the primary beam energy of the experiments i.e. 10 keV and the minimum d(hkl) threshold was kept at 0.5 Å. Using this reference Kikuchi pattern simulation, the gnomonic projections of the relevant zone axis were dynamically reprojected using the frameworks established in AstroEBSD [53] and MTEX [54, 55] in MATLAB R2023a environment. These electron channeling patterns were used to get the intensity of three orientations of ferroelastic domains, separated by a defined angle that follows their orientation relationship.

### 4.3 ECCI

A Zeiss Gemini SEM with a BSE detector was used for ECCI. To acquire sufficient signal from the ultrathin oxide films, a high gain was applied, and a 60 µm‐aperture in the high‐current mode was used for the incident electron beam at 9–10 kV. The sample was tilted by less than 5° and rotated in the plane to achieve the best contrast. The ECCI micrographs were processed in false colors with the Gwyddion image processing package for better visualization. In CASINO simulations, the energy of electron beam was set as 10 kV with a radius of 10 nm and a tilt of 3°. The density of SrRuO_3_, PbTiO_3_ and SrTiO_3_ were 6.46, 7.80, and 5.09 g cm^3^, respectively.

### 4.4 PFM Measurements

PFM measurements were performed with an Asylum MFP‐3D or Bruker Dimension Icon equipped with PPP‐EFM tips (Nanosensors). For PbTiO_3_ capacitors, the top SrRuO_3_ was patterned into circular geometries (with a diameter of 5 to 20 µm) using conventional photolithography and solvent etching by NaIO_4_ solution (0.1 M).

### 4.5 Device Fabrication and Transport Measurements

The transport of SrRuO_3_ (111) films was measured by patterning the films into a Hall bar geometry using conventional photolithograph and solvent etching by NaIO_4_ solution (0.1 M). The transverse resistivity was measured using a physical properties measurement system (Quantum Design).

### 4.6 Synchrotron Based Diffraction Measurements

Grazing incidence synchrotron‐based diffraction measurements were conducted using the ID28 diffractometer [56] at the European Synchrotron Radiation Facility, France with a PILATUS3 X 1M detector operated in shutterless mode at a sample to detector distance of 414 mm. An incident wavelength of 0.98 Å was used to avoid the L‐edge fluorescence of Pb, lower energy fluorescence was removed by a low energy threshold of 6.3 keV. All measurements were continuous, constant alpha, phi rotations conducted with the film rotated 52° perpendicular to the beam to improve reciprocal space coverage and avoid detector gaps. An effective grazing angle of 1.43° was chosen to keep the footprint of the beam inside the sample profile at all times as well as avoiding the critical edge of PbTiO_3_. Datasets were collected at two detector angles, 12° and 32°, and treated together for orientation matrix determination however the presented reconstruction is solely from the 12 degree dataset. The CrysAlis PRO software [57] was employed for orientation matrix determination and reciprocal space reconstructions were produced using custom in‐house software developed at the ID28 beamline.

### 4.7 DFT Calculations

DFT calculations were performed to investigate the magnetic and structural properties of bulk SrRuO_3_, using the Vienna Ab initio Simulation Package (VASP) [58]. The Perdew–Burke–Ernzerhof (PBE) form of the generalized gradient approximation (GGA) was employed for the exchange‐correlation functional [59]. To incorporate electron‐electron correlations beyond GGA, the DFT+U method was used with effective on‐site Coulomb and exchange parameters U = 3.0 eV and J = 0.3 eV, following the Liechtenstein formalism. The plane‐wave basis set was truncated at a kinetic energy cutoff of 500 eV, chosen through systematic convergence tests to ensure an optimal balance between computational efficiency and accuracy. The Brillouin zone was sampled using a Monkhorst‐Pack k‐point mesh, with sizes determined individually for different systems through convergence tests [60]. As SrRuO_3_ adopted an *Imma* structure with an oxygen octahedral tilting pattern of a^−^a^−^c^0^ while grown on an SrTiO_3_ (111) substrate [35], it was constructed an *Imma* primitive cell of 20 atoms including 4 Sr, 4 Ru and 12 O. For bulk calculations, a 7 × 5 × 7 k‐point mesh was used for Brillouin zone sampling. This calculations using DFT+U reveal that the energy per primitive cell with the spin along [100]*_pc_*, [001]*_pc_*, [110]*_pc_*, ${\left[1\bar{1}0\right]}_{pc}$ and [101]*_pc_* are −129.2446, −129. 2471, −129.2484, −129.2465 and −129.2467 eV, respectively. These results support the experimentally determined magnetic easy axis along the [110]*_pc_* direction [11].

To investigate the energetically favorable orientation of ferroelastic domain walls in SrRuO_3_ (111), we constructed supercells consisting of two ferroelastic domains based on the *Imma* primitive cell, the orientations of which were rotated by 120° with respect to each other. The supercell contains 240 atoms with 48 Sr, 48 Ru, 144 O. For the supercell calculations, a 2 × 5 × 4 mesh was applied for Brillouin zone sampling. Two different domain walls were considered: ${\left(\bar{1}10\right)}_{pc}$ and ${\left(11\bar{2}\right)}_{pc}$, both of which are perpendicular to the (111) plane. The resulting total energies were −1661.38 eV and −1661.40 eV per supercell, respectively, indicating that the domain wall in the ${\left(11\bar{2}\right)}_{pc}$ plane is thermodynamically more favorable. We did not consider other low‐index crystalline planes, e.g., (100)_pc_ and (110)*_pc_*, as they are inclined to the (111) plane and we did not observe any variation of contrast across the domain walls as those in the PbTiO_3_ films (Figure 2c; Figure S10).

## Conflicts of Interest

The authors declare no conflicts of interest.

## Supporting information




**Supporting File**: adma72216‐sup‐0001‐SuppMat.docx.

## Data Availability

The data that support the findings of this study are available from the corresponding author upon reasonable request.
