# Supplementary material for: Electron Channeling Contrast Imaging of Ferroelastic Domains
Source: Adv Mater. 2026 Jan 21;38(13):e15762. doi: 10.1002/adma.202515762 (PMC12957860; doi:10.1002/adma.202515762)
Supplement: Supplementary file 1 — Supporting File: adma72216‐sup‐0001‐SuppMat.docx. [file ADMA-38-e15762-s001.docx]

**Supporting Information**

**Supplementary figures**


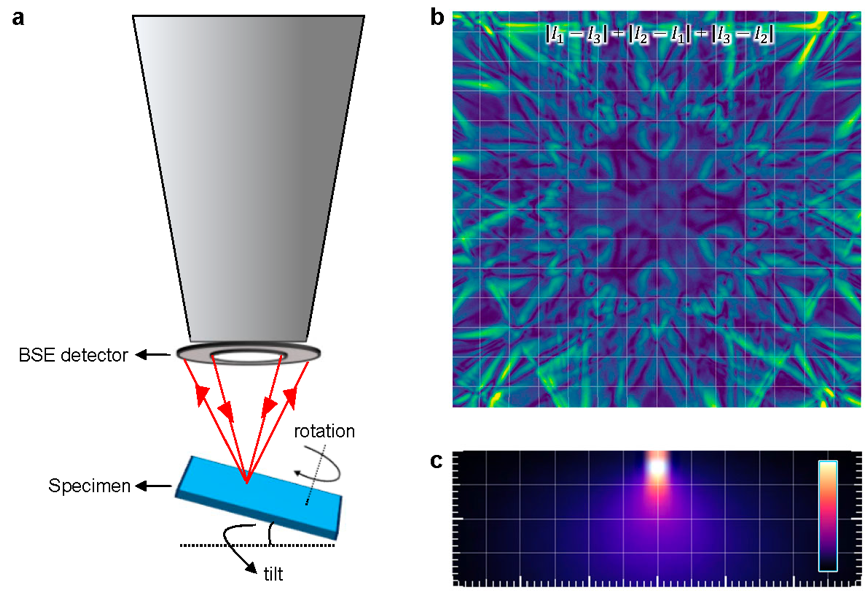


**Figure S1**. **a**) Geometry of the ECCI set-up, where the relative angle between the electron beam and the crystal is tuned by tilting and rotating the specimen. **b**) Three-domain contrast |*I*_1_- *I*_3_| + | *I*_1_- *I*_2_| + | *I*_2_- *I*_3_| (i.e., sum of absolute contrast differences in Figure 1c). Brighter regions indicate specimen orientations that maximize contrast between all three variants (see also supplementary interactive simulation in [10.5281/zenodo.17575062](https://doi.org/10.5281/zenodo.17575062)). The angular range is the same as Figure 1; overlaid mesh indicates specimen tilts of 1°. **c**) Monte-Carlo simulation of 10 keV electron interaction volume in bulk SrRuO_3_. Brighter color indicates a larger number of scattering events. Overlaid mesh is 50 nm.


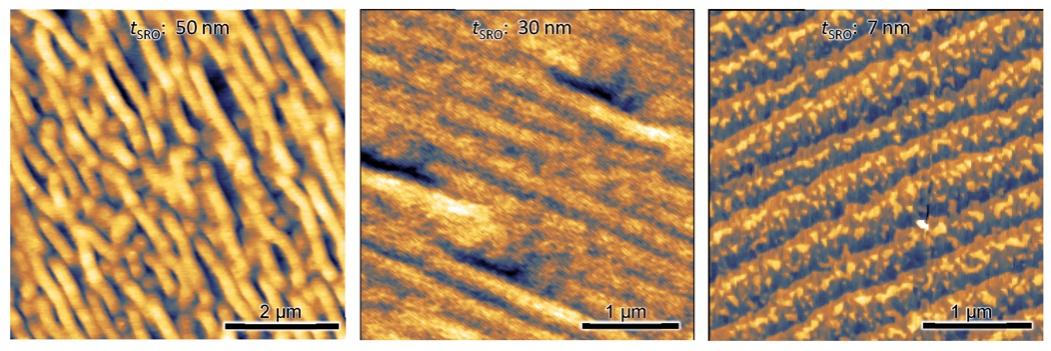


**Figure S2**. AFM topography of SrRuO_3_ (111) films with different thicknesses: 50, 30, and 7 nm. The topographic step terraces (with a height of unit cell, ~0.23 nm) in thinner films (≤30 nm) originate from substrate miscut, which match the stripe ferroelastic domains by ECCI in Fig. 2. The islands in the 7 nm-thick film also have a height of a unit cell, originated from incomplete growth of a layer during layer-by-layer growth.


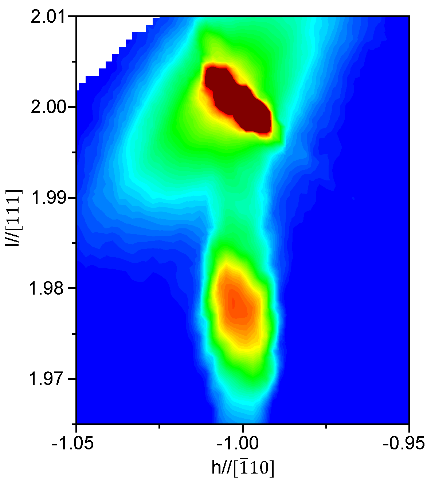


**Figure S3**. Asymmetric reciprocal space mapping of the (312) plane of SrRuO_3_/SrTiO_3_ (111) with a SrRuO_3_ thickness of 50 nm. The in-plane coherency between the film (lower peak) and the substrate (higher peak) suggests the fully strained state of the film.

**
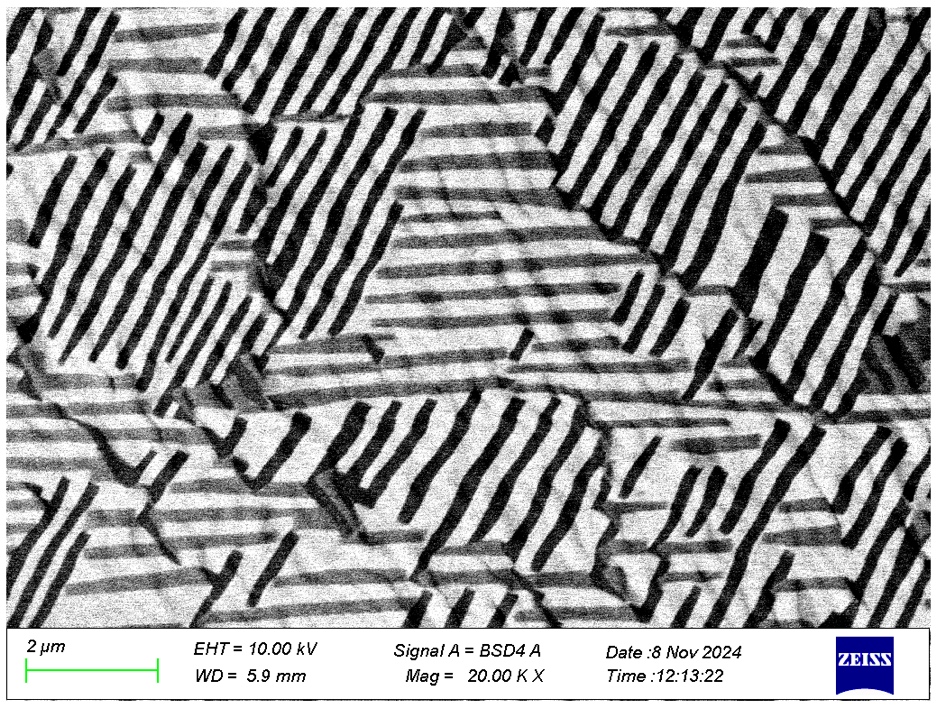
**

**Figure S4**. Original ECCI micrograph of Figure 2a in the main text.


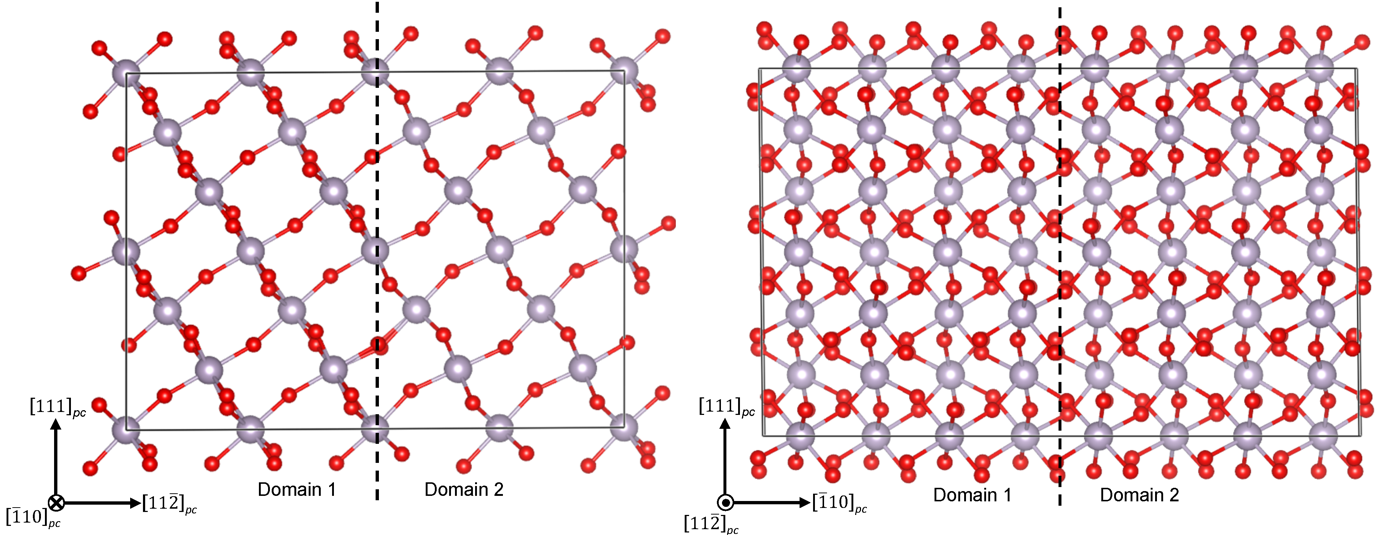


**Figure S5**. Two types of ferroelastic domain walls, i.e. ($11\bar{2}$) in left panel and ($\bar{1}10$) in the right panel, considered in our DFT calculations. Purple and red atoms are Ru and O, respectively. Sr atoms were omitted for simplicity.


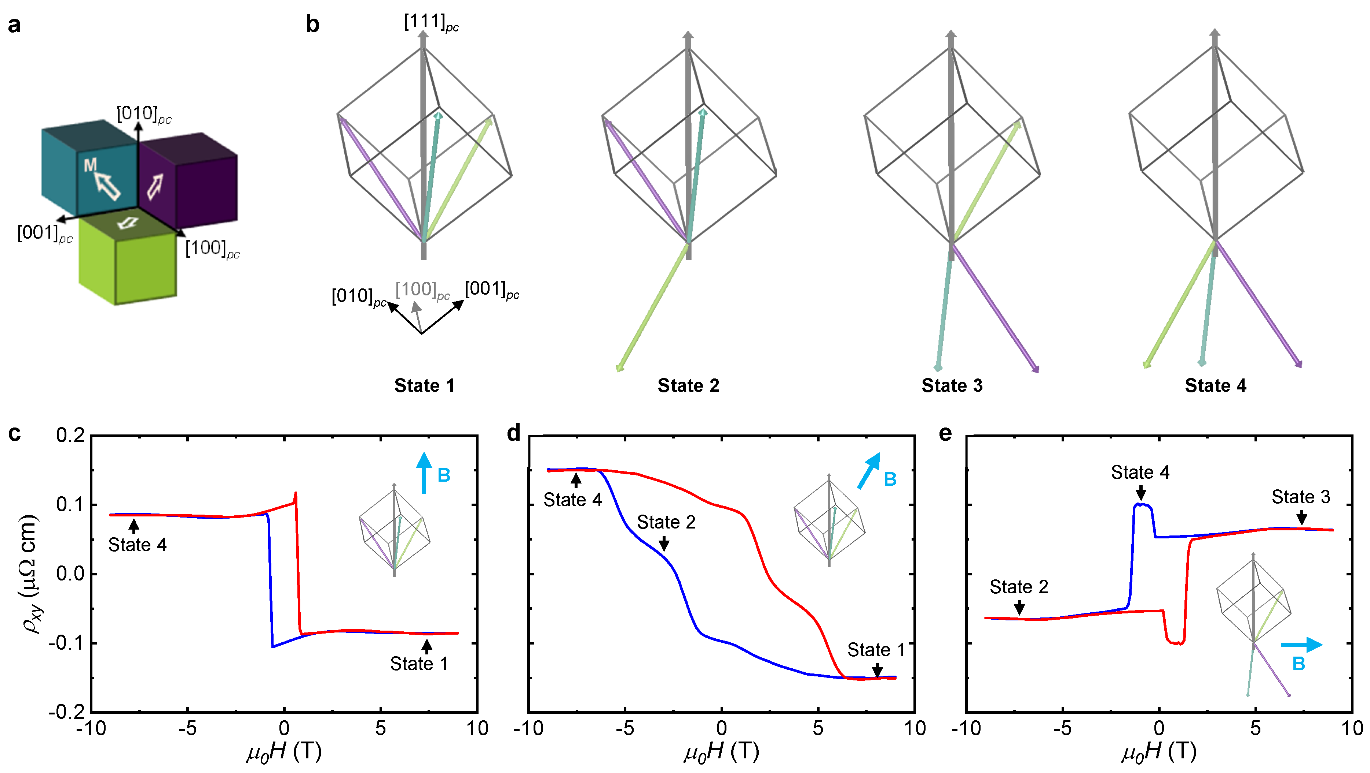


**Figure S6**. Magnetotransport study of the SrRuO_3_/SrTiO_3_ (111) heterostructure. **a**) Schematic of the magnetic easy axes (MEAs) of SrRuO_3_ with respect to its ferroelastic variants, which align along the pseudocubic face diagonal, namely <110>. **b**) Four possible configurations of magnetic dipoles of the ferroelastic domains, based on the ferroelastically coupled MEAs in the SrRuO_3_ (111) film. **c-e**) Anomalous Hall conductivity of the SrRuO_3_/SrTiO_3_ (111) heterostructure with a scanning magnetic field along the out-of-plane direction (c), tilted by 30° from the out-of-plane direction (d), and perpendicular to the out-of-plane direction (e). Depending on the relative direction between the magnetic field and the MEAs, the field component that is responsible for reversing the magnetic dipoles along each MEA is varied, resulting in different coercive fields and intermediate magnetic states.


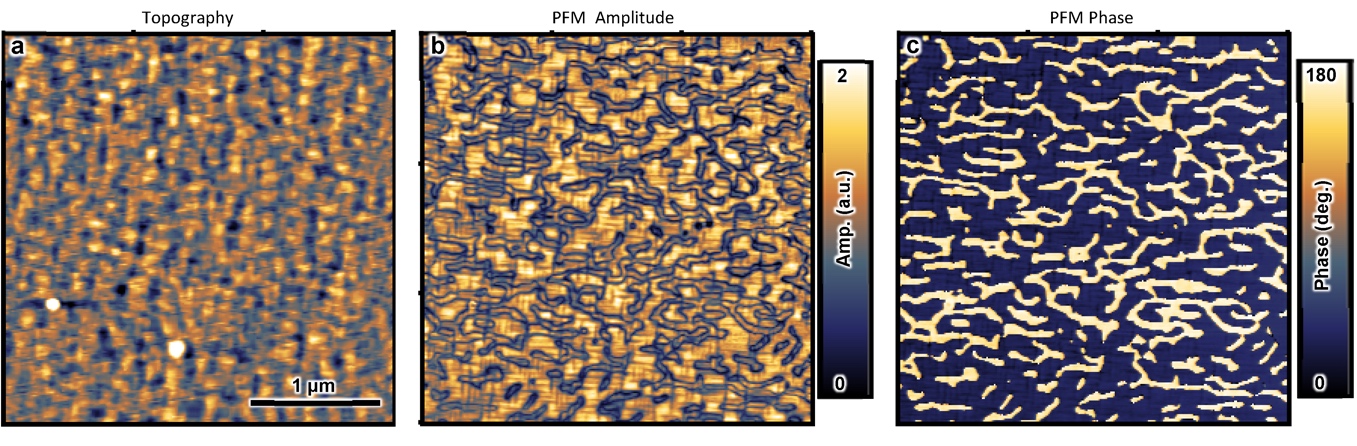


**Figure S7**. AFM topography (**a**) and out-of-plane PFM (**b** and **c**) images of a PbTiO_3_/DyScO_3_ (110) heterostructure. Besides the apparent c^+^/c^–^ 180° domains in both amplitude and phase images, there are also stripe mosaic patterns in the amplitude image, which correspond to the a/c domains.


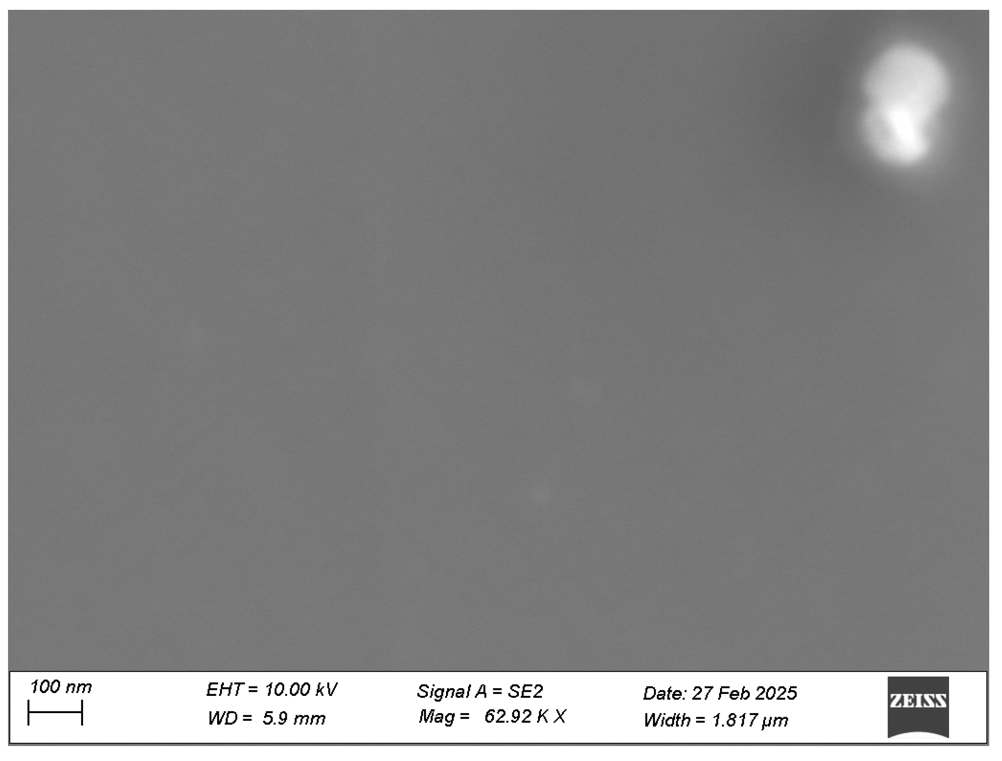


**Figure S8**. Secondary electron (SE) image of the PbTiO_3_/DyScO_3_ (110) heterostructure. Except topographic features of extrinsic contaminants on the top-right corner, there are no apparent features of the ferroelectric and ferroelastic domains, as those captured by PFM and ECCI.


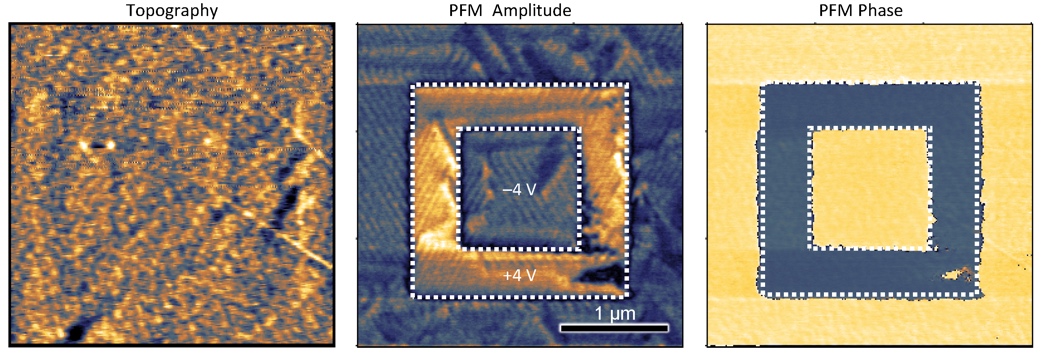


**Figure S9**. AFM topography (**a**) and out-of-plane PFM (**b** and **c**) images of a PbTiO_3_/SrRuO_3_/SrTiO_3_ (111) heterostructure. In the PFM images, box-in-box lithography was performed at –4 V for the inner box and 4 V for the outer box. The phase image suggests that the pristine film possesses uniform upward polarization.


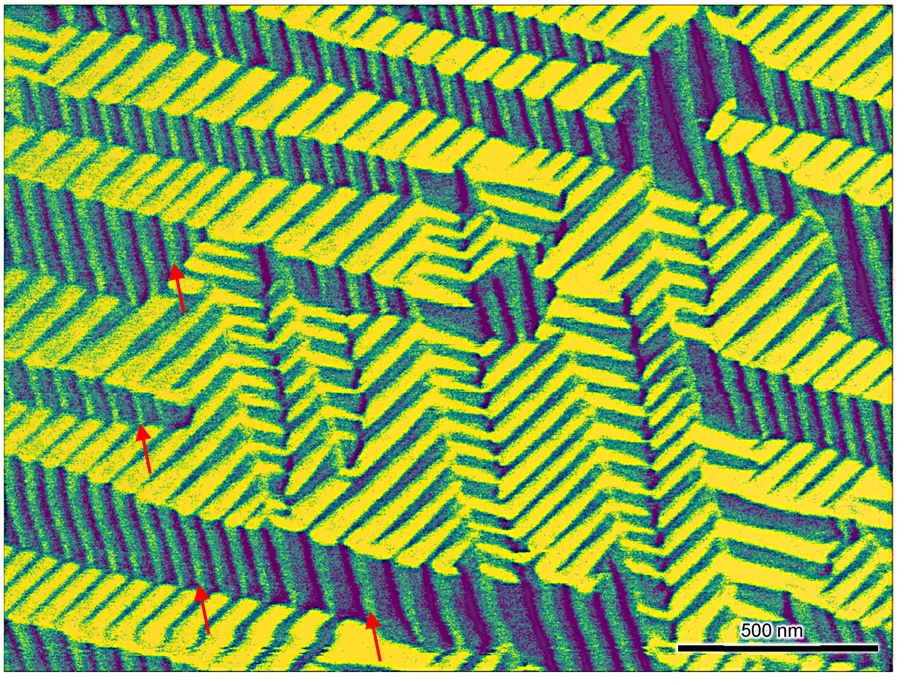


**Figure S10**. Higher-magnification ECCI micrograph of the PbTiO_3_ (~30 nm)/SrRuO_3_/SrTiO_3_ (111) heterostructure. Additional contrast variation across the domain walls as indicated by the red arrows may be caused by the inclined {110} domain walls.


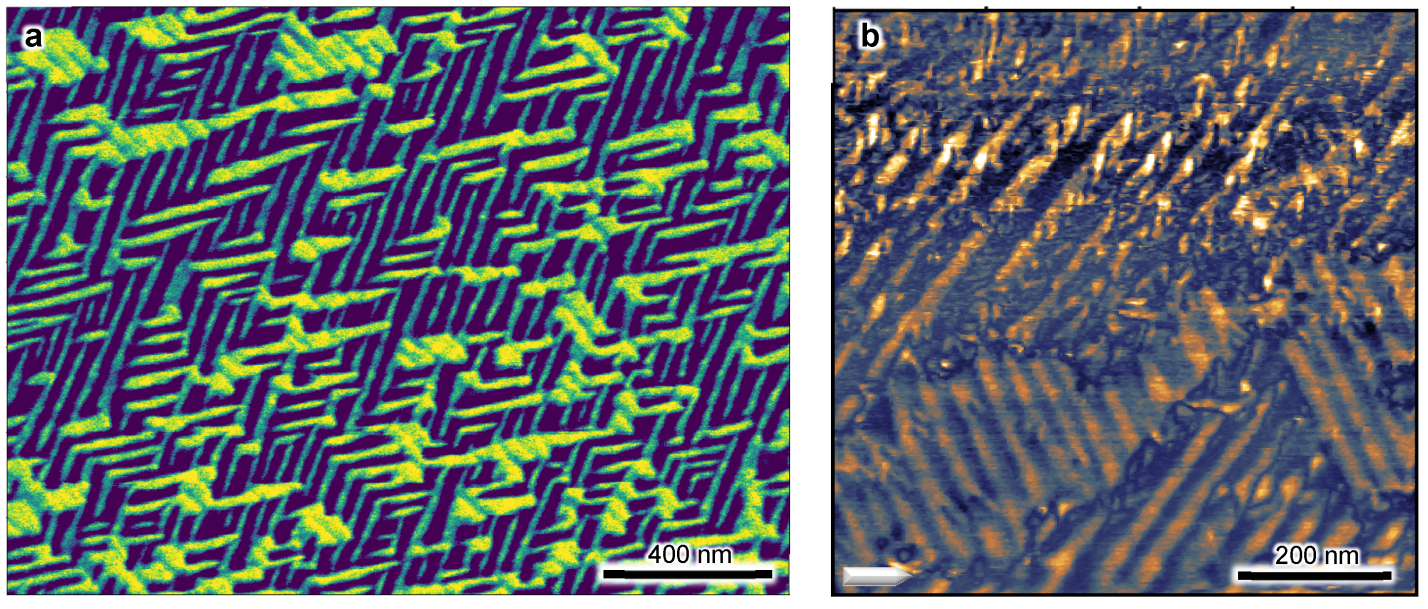


**Figure S11**. ECCI micrograph (**a**) and in-plane PFM image (**b**) of a PbTiO_3_/SrRuO_3_/SrTiO_3_ (111) heterostructure with a PbTiO_3_ thickness of ~6 nm, showing a domain periodicity of ~40 nm.


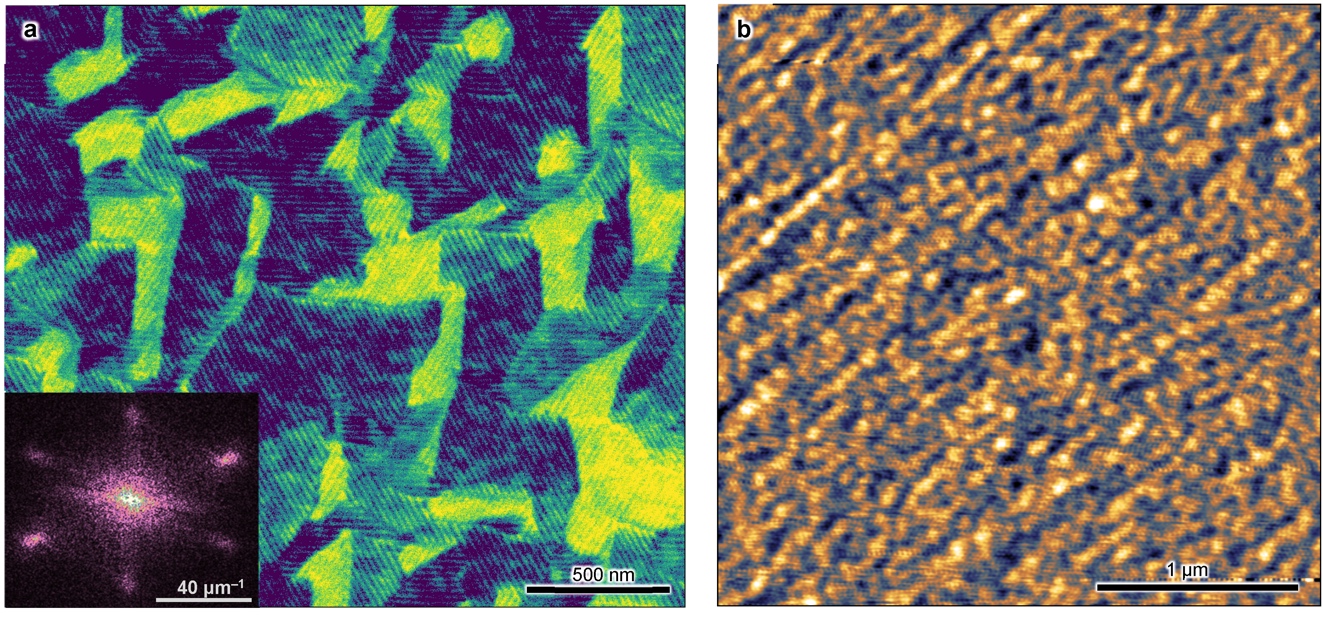


**Figure S12**. Low-magnification ECCI micrograph (**a**) and AFM topographic image (**b**) of the SrRuO_3_/PbTiO_3_ (30 nm)/SrRuO_3_/SrTiO_3_ (111) heterostructure as Fig. 4 in the main text. The topographic height variation is within 0.5 nm, indicating the smoothness. Inset of (a) shows the corresponding FFT of the ECCI micrograph. The domain periodicity was extracted to be approximately 22 nm.


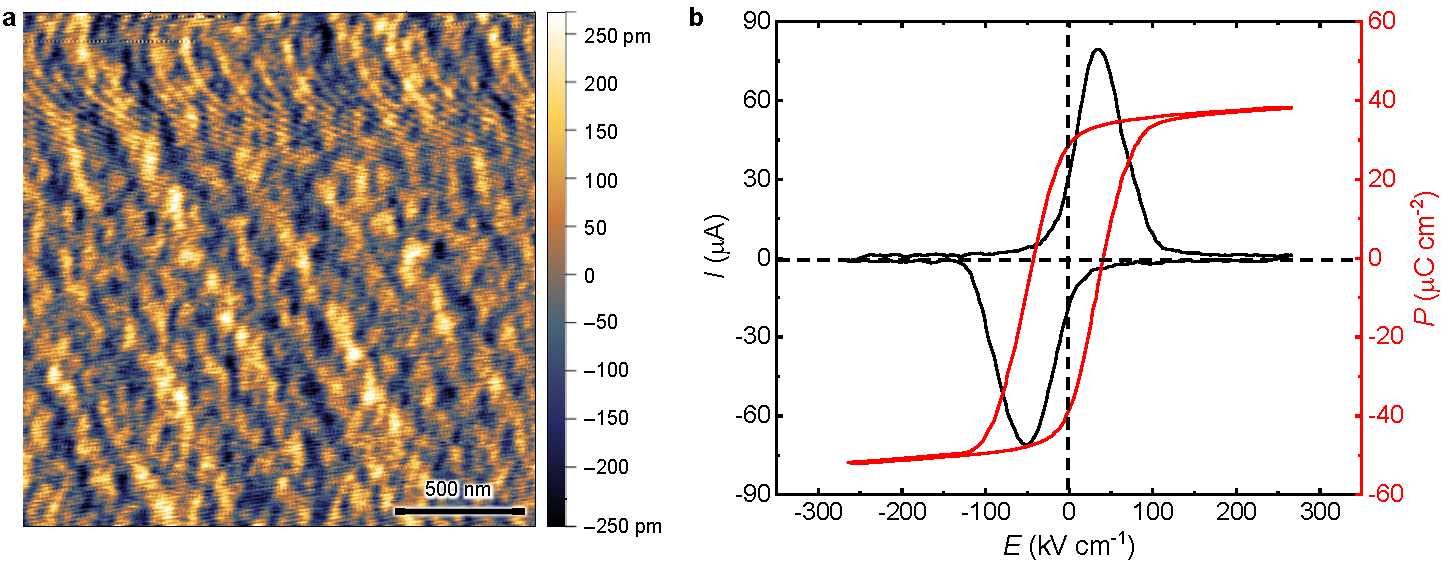


**Figure S13**. a) AFM topographic image and b) polarization hysteresis loop of the SrRuO_3_/PbTiO_3_ (10 nm)/SrRuO_3_/SrTiO_3_ (111) heterostructure.


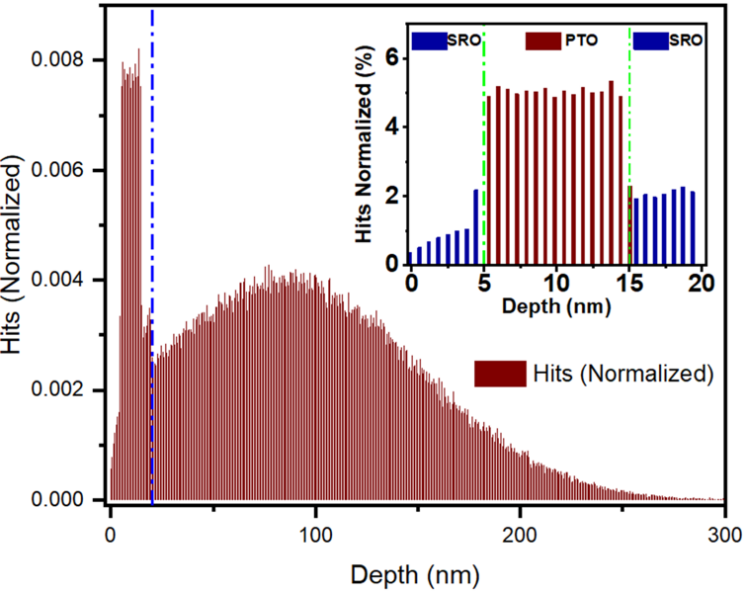


**Figure S14**. Simulation of backscattering signal from a SrRuO_3_ (5 nm)/PbTiO_3_ (10 nm)/SrRuO_3_ (5 nm)/SrTiO_3_ (111) heterostructure. The energy of the electron beam in the simulation was set as 10 kV with a radius of 10 nm and a tilt of 3°. The density of SrRuO_3_, PbTiO_3_ and SrTiO_3_ are 6.46, 7.80, and 5.09 g/cm^3^, respectively.


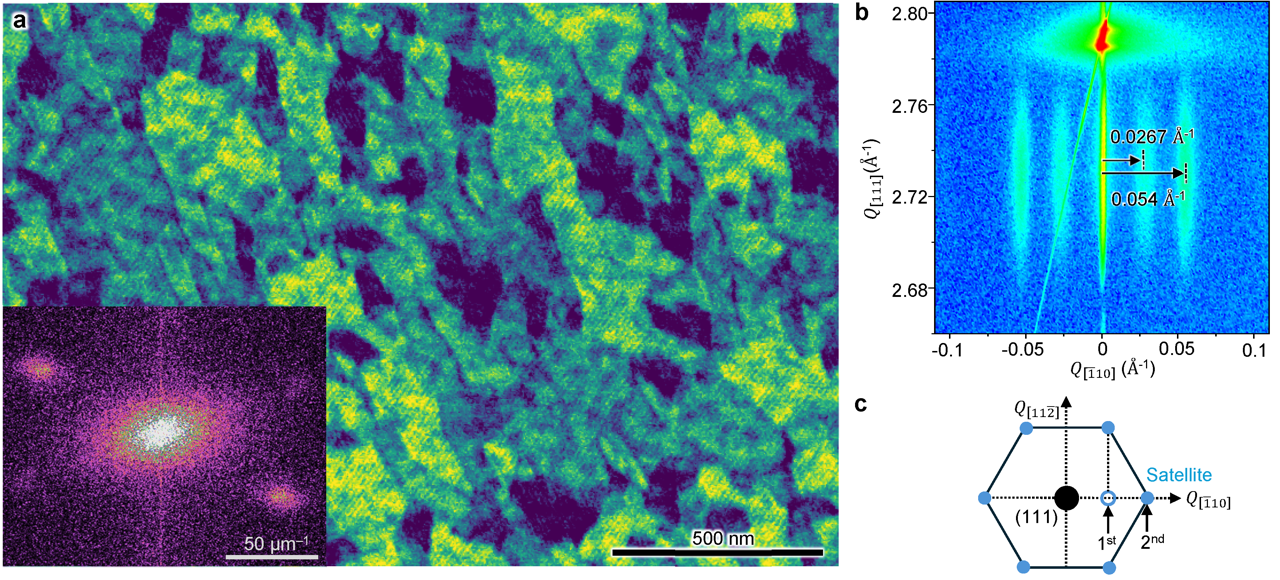


**Figure S15**. ECCI micrograph (**a**) and lab-based X-ray RSM (**b**) of a SrRuO_3_ (3 nm)/PbTiO_3_ (9 nm)/SrRuO_3_ (5 nm)/SrTiO_3_ (111) heterostructure. Inset of (a) shows the corresponding FFT image. c) Schematic of the satellite peaks in reciprocal space originating from periodic domains. The finite divergence of the X-ray beam in the lateral direction and the finite acceptance of the one-dimensional detector in our lab diffractometer leads to a lowering of the resolution perpendicular to the scattering plane and to projections of satellites onto this plane. Both methods reveal a domain periodicity of approximately 12 nm, corresponding to a domain width of approximately 6 nm.


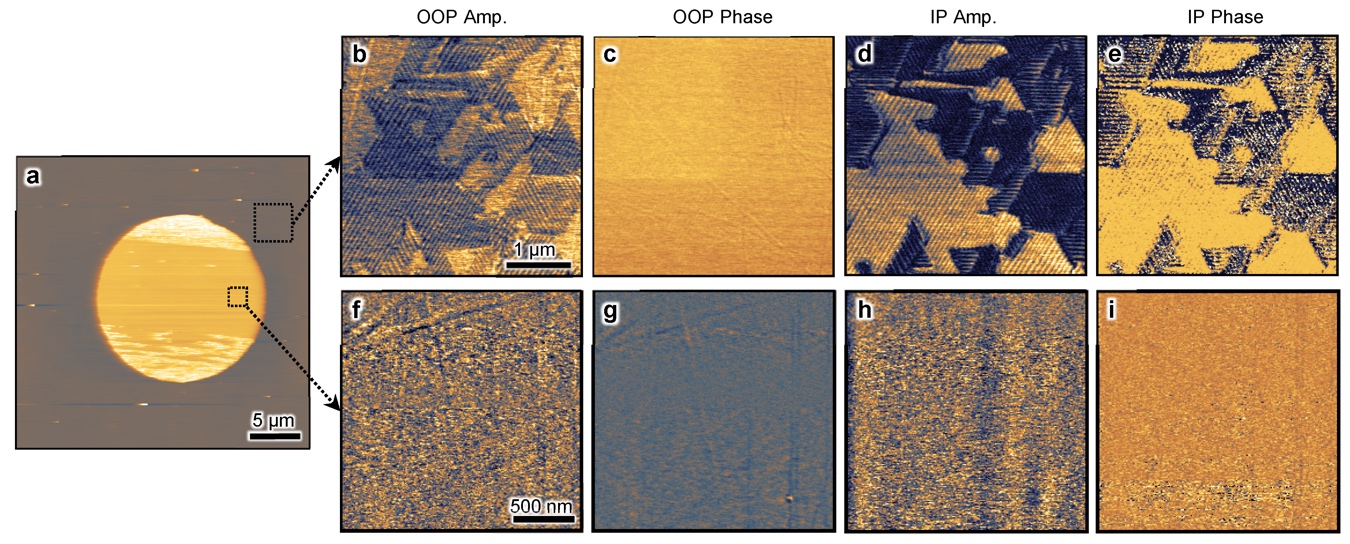


**Figure S16**. Vector PFM of the SrRuO_3_/PbTiO_3_ (30 nm)/SrRuO_3_/SrTiO_3_ (111) heterostructure with patterned top SrRuO_3_ electrode with a thickness of 3 nm. PFM on bare PbTiO_3_ shows clear domain structures with a periodicity of ~60–70 nm as observed in the simple PbTiO_3_/SrRuO_3_/SrTiO_3_ (111) heterostructure. However, PFM failed to resolve the domains on SrRuO_3_-capped region.
